# Interim 2025/26 LP.8.1 vaccine effectiveness estimates against COVID-19 from the Canadian Sentinel Practitioner Surveillance Network (SPSN): insights into possible impact of influenza and other respiratory virus co-circulation

**DOI:** 10.2807/1560-7917.ES.2026.31.18.2600331

**Published:** 2026-05-07

**Authors:** Danuta M Skowronski, Yuping Zhan, Samantha E Kaweski, Michelle B. Cox, Lea Separovic, Sara Carazo, Romy Olsha, Katie Dover, Suzana Sabaiduc, Christine Lacroix, Richard G Mather, Maan Hasso, Inès Levade, Agatha N Jassem, Isabelle Meunier, Ruimin Gao, Nathalie Bastien

**Affiliations:** 1 BC Centre for Disease Control, Vancouver, Canada; 2 University of British Columbia, Vancouver, Canada; 3 Institut national de santé publique du Québec, Québec, Canada; 4 Public Health Ontario, Toronto Canada; 5 Queen’s University, Kingston, Canada; 6 National Microbiology Laboratory, Public Health Agency of Canada, Winnipeg Canada

**Keywords:** COVID-19, SARS-CoV-2, vaccine effectiveness, test-negative design, whole genome sequencing, epidemiology, public health, vaccines

## Abstract

**BACKGROUND:**

The Canadian Sentinel Practitioner Surveillance Network routinely undertakes multiplex respiratory virus testing, vaccine effectiveness (VE) estimation by test-negative design (TND), and whole genome sequencing (WGS) of vaccine-targeted viruses.

**AIM:**

To estimate 2025/26 LP.8.1 VE against community-based COVID-19, including variant-specific, and explore the impact of other respiratory viruses among COVID-19 cases and/or controls.

**METHODS:**

Participants were ≥ 12-year-old outpatients presenting with acute respiratory illness between 26 October 2025 and 07 March 2026. COVID-19 vaccination information was registry-based. Primary TND analyses excluded influenza virus-infected controls. Sensitivity analyses explored inclusion and/or exclusion of influenza and other respiratory viral infections among COVID-19 cases and/or controls. WGS supported VE interpretation and variant-specific estimation.

**RESULTS:**

We included 3,802 participants (2,832 (74%) aged 12–64 years; 970 (26%) aged ≥ 65 years), with 310 COVID-19 cases (29 vaccinated; 9%) and 3,492 controls (577 vaccinated; 17%). At median 9 weeks post-vaccination, LP.8.1 VE was 48% (95%CI: 21 to 66): 44% (95%CI: −12 to 72) for 12–64 and 53% (95%CI: 21 to 73) for ≥ 65-year-olds. In sensitivity analyses, VE was stable for ≥ 65-year-olds. Among 12–64-year-olds, VE decreased when including influenza virus infections among controls but increased when excluding co-infections, recognising uncertainty with reduced sample size. Against viruses that failed vs succeeded WGS, VE was 26% (95%CI: −63 to 66) vs 53% (95%CI: 24 to 71), 63% (95%CI: 30 to 80) against the XFG variant. Most SARS-CoV-2 co-infections with semi-quantification, including those failing WGS, showed higher viral load for the non-SARS-CoV-2 infection.

**CONCLUSION:**

The 2025/26 LP.8.1 vaccine approximately halved the medically attended COVID-19 risk. Multiplex testing to identify primary co-infections among cases, or correlated vaccine-preventable infections among controls, may address VE under-estimation.

**Key public health message**

**What did you want to address in this study and why?**

Seasonal COVID-19 vaccination is recommended for people at increased risk of severe COVID-19, including ≥ 65-year-olds. During the 2025/26 respiratory virus season, the COVID-19 vaccine targeted the SARS-CoV-2 LP.8.1 strain but other variants circulated. The community-based Canadian Sentinel Practitioner Surveillance Network (SPSN) sought to estimate LP.8.1 VE against COVID-19 illness severe enough to require an outpatient medical visit.

**What have we learnt from this study?**

Within about 2 months since vaccination, the COVID-19 risk was approximately halved among people who got the LP.8.1 vaccine compared to those who did not. This includes protection against COVID-19 severe enough to require a doctor’s visit, and against illness due to SARS-CoV-2 variants other than LP.8.1. We also show how influenza and other respiratory viruses may in some circumstances lead to COVID-19 VE under-estimation, and how to address this issue.

**What are the implications of your findings for public health?**

The 2025/26 VE reported by the Canadian SPSN against medically attended outpatient COVID-19, including due to variants differing from the vaccine strain, is highly meaningful to individual protection. To realise the true benefits of immunisation programmes, methods to measure VE sometimes require adaptation depending upon the setting or context, which we illustrate to address the possible effects of other respiratory virus co-circulation.

## Introduction

As in recent prior seasons in North America and Europe, severe acute respiratory syndrome coronavirus 2 (SARS-CoV-2) activity peaked in early autumn ahead of the 2025/26 respiratory virus season, declining to mostly stable low levels thereafter [1-4]. Conversely, the 2025/26 influenza epidemic due to an antigenically drifted A(H3N2) variant called subclade K, peaked in late December or early January at levels rivalling historic maximums, disproportionately affecting youth [1-6]. A delayed but moderate peak in respiratory syncytial virus (RSV) circulation followed in mid- to late-February 2026 [1,2,4].

In Canada as elsewhere, seasonal COVID-19 vaccines are recommended for people aged ≥ 6 months who are at higher risk of severe COVID-19, with an age-based recommendation for all adults ≥ 65 years [7]. Autumn roll-out overlaps the influenza immunisation campaign in timing and certain target groups, with concurrent administration encouraged to reduce barriers to vaccine uptake. A spring COVID-19 booster dose, typically commencing in April, is also recommended in Canada for community-dwelling people who are moderately to severely immunocompromised or ≥ 80 years [7]. As recommended by the World Health Organization, updated COVID-19 vaccines for the 2025/26 season target the LP.8.1 strain [8], with two mRNA vaccines authorised in Canada for the autumn of 2025 [7].

The test-negative design (TND) was first pioneered for seasonal influenza vaccine effectiveness (VE) estimation against laboratory-confirmed influenza in 2004 [9], efficiently undertaken by comparing the odds of vaccination among patients who test positive (cases) versus negative (controls) for influenza virus through the adjusted odds ratio (OR), calculating VE as (1 − OR) × 100%. Since the COVID-19 pandemic, the TND has also been widely used for seasonal COVID-19 VE estimation. In 2022, Doll et al. first raised concern that correlated influenza and COVID-19 vaccination behaviours may bias VE estimates [10]. Such correlation can indirectly violate a core requirement for valid TND estimation, namely that the vaccine under evaluation must have no effect (direct or indirect) on other causes of respiratory illness included within the control series [11,12]. In the context of protective influenza vaccine, influenza virus infections within the COVID-19 control series are systematically less likely to be influenza vaccinated; with correlated vaccination they are also less likely to be COVID-19 vaccinated, potentially leading to COVID-19 VE under-estimation. Such bias, however, does not always operate or have meaningful VE impact. In simulations assuming relatively high influenza and COVID-19 vaccine coverages (> 50%), Doll et al. projected more influential bias with a higher proportion of influenza virus infections among COVID-19 controls (> 25%) and with higher influenza VE [10]. Despite widely recognised mismatch, in earlier interim analyses we and others reported moderate 2025/26 influenza VE estimates, albeit lower among older adults [13-15]. Given the substantial 2025/26 influenza epidemic and protective influenza vaccine, the role of influenza virus infections on 2025/26 COVID-19 VE estimation warrants consideration, typically addressed through exclusion from the COVID-19 control series. Recently, RSV vaccines have also been authorised, including since 2023 in Canada for pregnant people and adults ≥ 60 years. Although RSV, influenza and COVID-19 vaccines may be given concurrently, annual RSV re-vaccination is not currently recommended [16].

The Canadian Sentinel Practitioner Surveillance Network (SPSN) is a community-based respiratory virus and VE monitoring platform that routinely undertakes multiplex testing of respiratory specimens and whole genome sequencing (WGS) of contributing case viruses. In this TND analysis, the SPSN sought to estimate interim 2025/26 LP.8.1 VE against medically attended outpatient COVID-19, including age- and variant-specific estimates. We furthermore undertook empiric exploration of the impact of influenza, RSV and other respiratory viruses on VE estimation, including among COVID-19 cases to address differential exposure, vulnerability or severity risks associated with co-infections, and among controls to address potential bias associated with correlated vaccination behaviours.

## Methods

### Enrolment, specimen collection and testing

Sentinel practitioners collected respiratory specimens from consenting patients or their guardians presenting within 7 days of onset of acute respiratory illness (ARI, defined by new or worsening cough potentially due to respiratory infection). Participants within SPSN provinces of British Columbia (BC), Ontario and Quebec with specimen collection between 26 October 2025 and 07 March 2026 (epidemiological weeks (weeks) 44 to 09) are included. Accredited laboratories routinely applied molecular assays to SPSN specimens, including multiplex testing to detect SARS-CoV-2, influenza, RSV, seasonal coronaviruses, entero-/rhinoviruses (EV/RV), human metapneumovirus, adenovirus, and parainfluenza viruses, as listed by contributing SPSN province in Supplementary Table S1. Provincial laboratories and/or Canada’s National Microbiology Laboratory attempted WGS of case viruses, assigning SARS-CoV-2 lineage based on Pango nomenclature [17]. In BC and Quebec, WGS was attempted on all SARS-CoV-2 case viruses; in Ontario WGS was limited to specimens with sufficient viral load indicated through semi-quantification based upon quantification cycle (Cq) values < 30.

### Vaccine status ascertainment

Provincial start dates for the 2025/26 COVID-19 immunisation campaign ranged between weeks 39 and 41. In BC and Ontario, COVID-19 vaccination was publicly funded for anyone ≥ 6 months old, but in Quebec individuals other than high-risk priority groups and adults ≥ 65 years old had to pay as of the 2025/26 campaign. Influenza vaccination was publicly funded for anyone aged ≥ 6 months while RSV vaccine was universally funded for community-dwelling adults ≥ 75-years old in Ontario, but not in BC or Quebec.

Sentinel practitioners obtained self-reported influenza, COVID-19 and RSV vaccination status from patients at specimen collection, but consent and additional authorisation enabled subsequent extraction of COVID-19 vaccination details from provincial immunisation registries (PIR) in all SPSN provinces, except Alberta. Here, COVID-19 VE analyses presented use PIR-based vaccination status and are thus restricted to BC, Ontario and Quebec. Most COVID-19 immunisation data are entered directly into the PIR at the time of administration by the healthcare professional but delayed batch entry may occur for some settings. In Quebec, anyone with a health insurance number (vaccinated or unvaccinated) is registered within the COVID-19 PIR whereas in Ontario and BC, only people who have ever received a COVID-19 vaccine are captured. Participants without PIR documentation are considered unvaccinated by default. For the current analyses, COVID-19 vaccination status was extracted from the PIR during epidemiological weeks 10 or 11 of 2026.

### Vaccine effectiveness estimation

We estimated VE against medically attended outpatient ARI due to laboratory-confirmed COVID-19 by TND, excluding participants aged < 12 years owing to more complex COVID-19 vaccine dosing recommendations [7]. Vaccination status was defined by receipt of 2025/26 LP.8.1 vaccine ≥ 2 weeks before illness onset, excluding individuals vaccinated < 2 weeks prior; COVID-19 cases were SARS-CoV-2 test-positive and controls were SARS-CoV-2 test-negative. The VE was assessed overall and by age groups 12–64 and ≥ 65 years with the following conditions in primary analyses, modified in sensitivity analyses as specified:

(i) Adjustment for age group (12–49, 50–64, ≥ 65 years), province (BC, Ontario, Quebec), and calendar time (bi-weekly). Sensitivity analyses applied finer age groups and adjustment for sex, and comorbidity, excluding those with unknown information.

(ii) Without regard to prior COVID-19 vaccination history. Sensitivity analyses excluded 2025 spring booster-dose recipients and those with no PIR record of any COVID-19 doses at any time.

(iii) Retaining co-infections among COVID-19 cases. Sensitivity analyses excluded influenza, RSV and other viral co-infections.

(iv) Excluding influenza virus infections from COVID-19 controls. Sensitivity analyses retained influenza virus infections in controls, with and without including co-infections among COVID-19 cases and further excluded RSV and other viral infections from controls.

We also explored VE restricted to patients meeting a more severe influenza-like illness (ILI) syndrome inclusive of cough plus fever, feverishness or chills and one or more of the following symptoms: sore throat, myalgia, arthralgia or prostration. Finally, we explored VE estimates stratified by WGS findings, including variant-specific VE, all relative to the same control group as in primary analyses.

## Results

### SARS-CoV-2 and other respiratory virus detections

**Figure 1:**
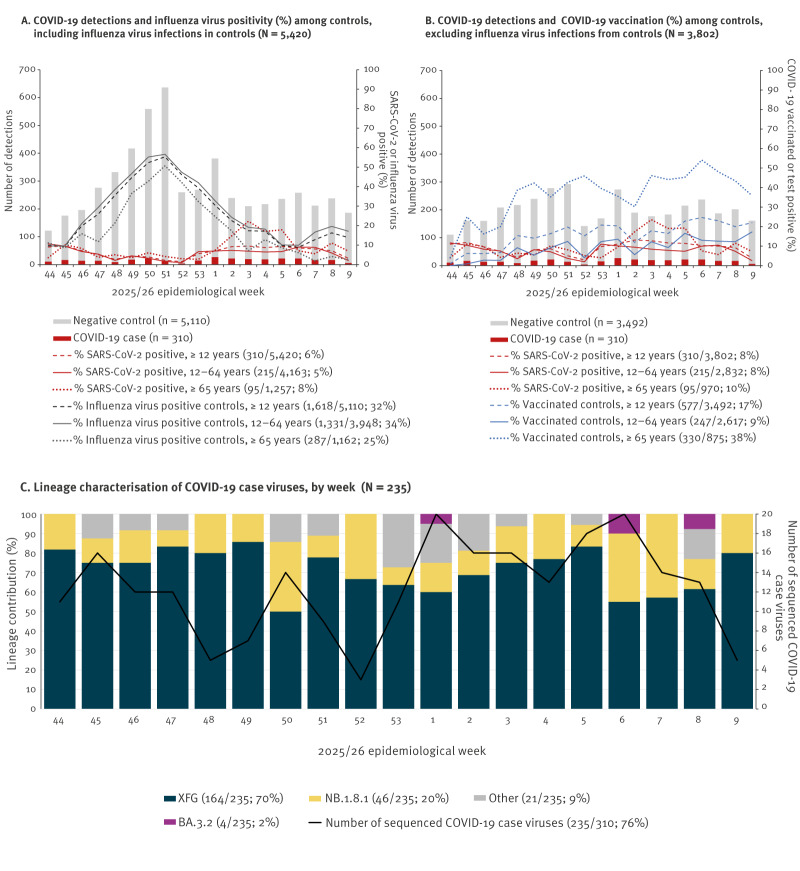
(A, B) Laboratory-confirmed COVID-19 and influenza, and COVID-19 vaccination curves, by week and age group (n = 5,420 cases and controls) and (C) lineage characterisation among sequenced viruses from confirmed COVID-19 cases (n = 235) percentage by week, Canadian Sentinel Practitioner Surveillance Network (SPSN), Canada, 26 October 2025–07 March 2026 (weeks 44–09)

PIR: provincial immunisation registry; SARS-CoV-2: severe acute respiratory syndrome coronavirus 2; VE: vaccine effectiveness.

Between weeks 44 and 09, 5,501 specimens were collected from ≥ 12-year-olds with known SARS-CoV-2 test results. Excluding 81 (1%) vaccinated < 2 weeks before illness onset left 5,420 contributing specimens including 310 (6%) COVID-19 cases and 5,110 controls (Figure 1A**)**.

Findings are displayed by epidemiological week of specimen collection restricted to participants included in primary PIR-based VE analyses (i.e. age ≥ 12 years from British Columbia, Ontario and Quebec, vaccinated ≥ 2 weeks before illness onset). **Panel A** retains influenza virus infections among COVID-19 controls and displays the per cent SARS-CoV-2 positive among participants and the per cent influenza virus positive among COVID-19 controls, overall and by age group. Influenza virus positivity curves do not include influenza virus infections among COVID-19 cases (n = 21 co-infections). **Panel B** applies the same restrictions but excludes influenza virus infections (n = 1,618) from the COVID-19 controls, as per primary VE analyses. Shown are the weekly per cent SARS-CoV-2 positive and the per cent of COVID-19 controls vaccinated ≥ 2 weeks before illness onset, overall and by age group. **Panel C** shows the weekly tally of sequenced COVID-19 case viruses and per cent lineage contribution by week among case viruses contributing to primary VE analyses.

Other respiratory virus contribution is shown in Supplementary Table S2. Among COVID-19 cases, 11% (35/310) were infected with another respiratory virus of which around two-thirds (21/35) were influenza. Overall, influenza virus co-infections comprised 7% (21/310) of COVID-19 cases: 18/215 (8%) among 12–64-year-olds and 3/95 (3%) among ≥ 65-year-olds. Of the 15 other co-infections, which included one case co-infected with SARS-CoV-2, influenza and a non-influenza virus, most (11/15) were also in 12–64-year-olds.

Among COVID-19 controls, ≥ 1 respiratory pathogen was identified in 60% (3,041/5,110), with 53% (1,618/3,041) being influenza viruses (Supplementary Table S2). Influenza virus infections represented nearly one-third of controls (1,618/5,110), higher for 12–64 (1,331/3,948; 34%) than ≥ 65-year-olds (287/1,162; 25%) (Figure 1A). Weekly influenza virus positivity among controls peaked at > 50% in weeks 50 and 51, exceeding 30% between weeks 48 and 01, when 77% (1,244/1,618) of influenza virus infections and more than half of COVID-19 controls accrued (Figure 1A), as further enumerated in Supplementary Table S3. Other respiratory viruses among controls mainly included EV/RV (535/5,110; 10%), RSV (349/5,110; 7%), and seasonal coronaviruses (263/5,110; 5%). With restriction to unvaccinated participants, seasonal coronavirus positivity overall (208/4,655; 4%) or among COVID-19 controls (206/4,374; 5%) was slightly lower than COVID-19 positivity (281/4,655; 6%) (p < 0.001). Excluding influenza virus infections (n = 1,618) from controls, we include 3,802 participants in primary VE analyses, 310 (8%) COVID-19 cases and 3,492 controls (Figure 1B).

### Whole genome sequencing of case viruses

We sequenced 76% (235/310) of contributing COVID-19 case viruses (GISAID Epi Set ID: EPI_SET_260424qa). There were 74 case viruses that failed WGS including 45 in Ontario for which it was not attempted due to high Cq. The XFG lineage predominated among sequenced viruses (70%; 164/235), followed by NB.1.8.1 (20%; 46/235), with no LP.8.1 (Figure 1C). Beginning week 1, Ontario identified four cases (4/157; 3%) of the BA.3.2 variant. Lineage and sub-lineage details by province, including those captured within the ‘other’ category displayed in Figure 1C, are shown in Supplementary Table S4.

### Participant characteristics

One-quarter (970/3,802) of participants were ≥ 65 years old, more among cases (95/310; 31%) than controls (875/3,492; 25%) (p = 0.03) (Table). Overall, 9% (29/310) of cases vs 17% (577/3,492) of controls were LP.8.1 vaccinated ≥ 2 weeks before illness onset (p < 0.001): 4% (9/215) vs 9% (247/2,617) of 12–64 (p < 0.01) and 21% (20/95) vs 38% (330/875) of ≥ 65-year-olds (p = 0.001), respectively (Figure 1B, Table). Over 90% of vaccinated participants received their LP.8.1 dose by end of November (week 48).

**Table:** Participant characteristics, excluding influenza virus infections from controls, Canadian Sentinel Practitioner Surveillance Network (SPSN), Canada, 26 October 2025–07 March 2026 (weeks 44–09) (n = 3,802)

Characteristics		All ARI participants^a^ (column %, unless otherwise specified)						COVID-19 vaccinated, PIR-based^b,c^ (row %, unless otherwise specified)					
		Overall		COVID-19 cases		COVID-19 controls		Overall		COVID-19 cases		COVID-19 controls	
		Number	%	Number	%	Number	%	Number	%	Number	%	Number	%
**Number (row %)**		**3,802**	**100**	**310**	**8**	**3,492**	**92**	**606**	**16**	**29**	**9**	**577**	**17**
**Age group (years)**													
12–49		1,997	53	157	51	1,840	53	138	7	5	3	133	7
50–64		835	22	58	19	777	22	118	14	4	NC	114	15
65–79		742	20	83	27	659	19	253	34	15	18	238	36
≥ 80		228	6	12	4	216	6	97	43	5	NC	92	43
Median (IQR)		48 (33–65)		48.5 (34–68)		48 (33–65)		67 (52–76)		72 (63–77)		67 (52–75)	
**Sex**													
Female		2,467	65	210	68	2,257	65	384	16	18	9	366	16
Male		1,323	35	99	32	1,224	35	221	17	11	11	210	17
Unknown		12	0	1	0	11	0	1	NC	0	NC	1	NC
**Comorbidity^d^**													
No		2,486	65	200	65	2,286	65	318	13	17	9	301	13
Yes		973	26	79	25	894	26	240	25	9	11	231	26
Unknown		343	9	31	10	312	9	48	14	3	NC	45	14
**Province**													
British Columbia		910	24	28	9	882	25	228	25	4	NC	224	25
Ontario		1,979	52	222	72	1,757	50	247	12	14	6	233	13
Quebec		913	24	60	19	853	24	131	14	11	18	120	14
**Period of specimen collection (epidemiological week span), 2025/26^e^**													
44–47		640	17	56	18	584	17	34	5	2	NC	32	5
48–01		1,610	42	109	35	1,501	43	277	17	14	13	263	18
02–09		1,552	41	145	47	1,407	40	295	19	13	9	282	20
**COVID-19 spring 2025 vaccination status (PIR-based; column %)^f^**													
12–64-year-olds	**Total number**	**2,832**	**%**	**215**	**%**	**2,617**	**%**	**256**	**%**	**9**	**%**	**247**	**%**
	Yes	13	< 1	0	0	13	< 1	9	4	0	NC	9	4
	No	2,819	100	215	100	2,604	100	247	96	9	NC	238	96
≥ 65-year-olds	**Total number**	**970**	**%**	**95**	**%**	**875**	**%**	**350**	**%**	**20**	**%**	**330**	**%**
	Yes	114	12	7	7	107	12	76	22	4	NC	72	22
	No	856	88	88	93	768	88	274	78	16	NC	258	78
**Seasonal 2025/26 influenza vaccination status (sentinel-based; column %)^g^**													
12–64-year-olds^h^	**Total number**	**2,832**	**%**	**215**	**%**	**2,617**	**%**	**256**	**%**	**9**	**%**	**247**	**%**
	Yes	624	22	35	16	589	23	225	88	8	NC	217	88
	No	1,990	70	159	74	1,831	70	19	7	1	NC	18	7
	Unknown	218	8	21	10	197	8	12	5	0	NC	12	5
≥ 65-year-olds^i^	**Total number**	**970**	**%**	**95**	**%**	**875**	**%**	**350**	**%**	**20**	**%**	**330**	**%**
	Yes	508	52	46	48	462	53	275	79	14	NC	261	79
	No	378	39	39	41	339	39	48	14	4	NC	44	13
	Unknown	84	9	10	11	74	8	27	8	2	NC	25	8

ARI: acute respiratory illness; CI: confidence interval; IQR: interquartile range; NC: not calculated (denominator < 60); OR: odds ratio; PIR: provincial immunisation registry; VE: vaccine effectiveness.

^a^ Participants as per primary VE analyses: aged ≥ 12 years from British Columbia, Ontario and Quebec; excluding influenza test-positive cases from COVID-19 controls.

^b^ Vaccination status PIR-based and refers to LP.8.1 vaccination as part of autumn 2025/26 COVID-19 immunisation campaign, received ≥ 2 weeks before illness onset. Participants without PIR documented record of having received the specified vaccine are considered unvaccinated, including those with no PIR record of COVID-19 vaccine receipt at any time since the pandemic (n = 333); those vaccinated since < 2 weeks are excluded (n = 58). Only specimens confirmed by the submitting sentinel to have been collected within 7 days of ARI onset are included. Accordingly, where ARI onset date is missing, specimens collected ≥ 21 days after vaccination date are considered to have been vaccinated ≥ 2 weeks before ARI onset (n = 9). Conversely, if ARI onset date is missing and the interval between vaccination and specimen collection date is < 2 weeks then vaccination is assigned as < 2 weeks before onset (n = 1).

^c^ Based on PIR data, earliest LP.8.1 vaccination (any timing or ≥ 2 weeks before illness onset) was week 39 in Ontario, week 41 in British Columbia and week 42 in Quebec. Overall whether based on any timing or ≥ 2 weeks before onset, > 90% were vaccinated by the end of week 48.

^d^ Includes chronic comorbidities increasing risk of serious influenza complications as per Canada's National Advisory Committee on Immunisation [33].

^e^ Missing specimen collection dates were imputed as the date the specimen was received and processed at the laboratory minus 2 days. In VE analyses, calendar time adjustment undertaken bi-weekly (except weeks 07 to 09).

^f^ Defined as participants (cases or controls) KP.2 vaccinated between 01 April 2025 and 30 September 2025 (British Columbia or Quebec) or 01 April 2025 and 17 September 2025 (Ontario), as per PIR documentation. Last recorded date of receipt was June 2025 in British Columbia, July 2025 in Ontario, and August 2025 in Quebec.

^g^ Sentinel-based report of 2025/26 seasonal influenza vaccination without regard to timing in relation to illness onset.

^h^ OR, influenza vaccination (yes vs no/unknown) for COVID-19 vaccinated (217/247; 88%) vs unvaccinated (372/2,370; 16%) controls: 38.9 (95%CI: 26.1 to 57.8), similar but more pronounced when excluding those with unknown self-reported influenza vaccination status.

^i^ OR, influenza vaccination (yes vs no/unknown) for COVID-19 vaccinated (261/330; 79%) vs unvaccinated (201/545; 37%) controls: 6.5 (95%CI: 4.7 to 8.9), similar but more pronounced when excluding those with unknown self-reported influenza vaccination status.

In the 2024/25 Canadian national coverage survey, 26% of ≥ 18-years-olds and 54% of ≥ 65-years-olds self-reported COVID-19 vaccination [18], with updated estimates for 2025/26 pending. In our 2024/25 mid-season analysis (weeks 44 to 09), 20% of ≥ 18-year-old and 43% of ≥ 65-year-old controls (excluding influenza virus infections) had PIR documentation of vaccination (without regard to timing before symptom onset), increasing slightly to 22% and 45%, respectively, by end-of-season [19]. For the current 2025/26 analysis (weeks 44 to 09) applying the same criteria, 19% of ≥ 18-year-old and 40% of ≥ 65-year-old controls were vaccinated, generally consistent if slightly lower than 2024/25. Although not yet publicly available from all provinces for 2025/26, available provincial population-based coverage estimates generally align with the proportion vaccinated within our SPSN control series [20].

Overall, 3,019/3,802 (79%) ARI participants presented with ILI, similar among 12–64 (2,242/2,832; 79%) and ≥ 65-year-olds (777/970; 80%) (p = 0.53). The proportion with ILI was higher for cases than controls among participants aged 12–64 years (188/215; 87% vs 2,054/2,617; 78%) (p = 0.002) but not ≥ 65 years (77/95; 81% vs 700/875; 80%) (p = 0.81).

Participant profiles were similar whether excluding (Table) or including (Supplementary Table S3) influenza virus infections among controls. With inclusion there was slight reduction in the median age of controls (48 vs 45 years) and the proportion of controls vaccinated (17% vs 14%). In both series, < 1% of 12–64 and < 15% of ≥ 65-year-olds had a PIR record of 2025 spring booster-dose receipt (< 5% and < 25%, respectively, among 2025/26 LP.8.1-vaccinated participants).

Per sentinel reporting, 22% of 12–64 and 52% of ≥ 65-year-olds included in primary VE analyses indicated they had received the 2025/26 influenza vaccine (24% and 57%, respectively, excluding those with unknown status), and higher at > 85% and > 75%, respectively, among LP.8.1-vaccinated participants. Among controls, 2025/26 influenza and COVID-19 vaccinations were associated, more strongly among 12–64 (OR: 38.9; 95% confidence interval (CI): 26.1 to 57.8) than ≥ 65-year-olds (OR: 6.5; 95%CI: 4.7–8.9), similar but more pronounced excluding those with unknown influenza vaccination status and/or when influenza virus infections were retained among controls (Supplementary Table S3). Conversely, just 15% (141/970) of ≥ 65-year-olds, and 21% (74/350) who were COVID-19 vaccinated, self-reported any RSV vaccination (141/817; 17% and 74/291; 25%, respectively, excluding those with unknown status).

### Vaccine effectiveness findings

**Figure 2:**
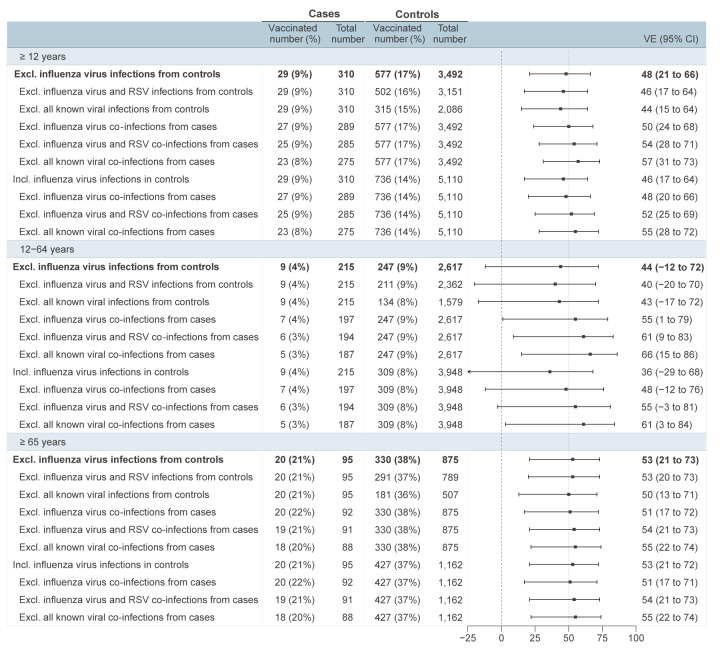
2025/26 COVID-19 VE estimates, primary and sensitivity analyses varying other respiratory virus exclusion criteria, overall and by age group, Canadian Sentinel Practitioner Surveillance Network (SPSN), Canada, 26 October 2025–07 March 2026 (weeks 44–09) (n = 3,802)

In primary analyses, adjusted COVID-19 VE at median 9 weeks post-vaccination was 48% (95%CI: 21 to 66): 44% (95%CI: −12 to 72) among 12–64-year-olds and 53% (95%CI: 21 to 73) among ≥ 65-year-olds (Figure 2). Complete time since vaccination details accompanying VE estimates are shown in Supplementary Figure S1.

CI: confidence interval; Excl.: excluding; Incl.: including; RSV: respiratory syncytial virus; SARS-CoV-2: severe acute respiratory syndrome coronavirus 2; VE: vaccine effectiveness.

Primary VE analyses are shown in bold. Primary analyses are without regard to prior COVID-19 vaccination or infection history (including 2025 spring vaccinated) and exclude influenza virus infections from COVID-19 controls (retaining influenza co-infections among COVID-19 cases). Sensitivity analyses explore VE by modifying inclusion or exclusion criteria related to influenza, RSV and other respiratory virus infections among COVID-19 cases and/or controls.

Analyses are restricted to participants ≥ 12 years from the provinces of British Columbia, Ontario or Quebec. Current vaccination status is based upon provincial immunisation registry documentation of 2025/26 LP.8.1 COVID-19 vaccine receipt. VE is assessed at ≥ 2 weeks post-vaccination; participants vaccinated < 2 weeks before illness onset excluded. All VE estimates are adjusted for age group (12–49, 50–64, ≥ 65 years), province (British Columbia, Ontario, Quebec), and calendar time (based upon specimen collection date, bi-weekly except weeks 07 to 09). Control specimens with indeterminate results for any non-SARS-CoV-2 viral targets and those positive for bocavirus or atypical bacteria (because not assessed in Ontario) are retained throughout (Supplementary Tables S1 and S2).

In sensitivity analyses, all VE estimates remained within 3% of primary when modifying covariate adjustment as depicted in Supplementary Figure S2, excluding 2025 spring booster-dose recipients, or participants with no COVID-19 PIR record, as shown in Supplementary Figure S3. 

In other sensitivity analyses to explore the impact of other respiratory viruses among COVID-19 cases or controls, VE estimates were stable for ≥ 65-year-olds, also remaining within 3% of primary analyses. While 12–64-year-olds were more affected by other respiratory viruses, the number of vaccinated cases was lower and CIs wider, resulting in potentially greater instability. The VE point estimates for 12–64-year-olds remained within 4% of primary when excluding not only influenza, but also RSV and other respiratory viruses from controls. Conversely, VE point estimates among 12–64-year-olds steadily increased with incremental exclusion of influenza (+11%), RSV (+17%) and finally all co-infections (+22%) from cases (Figure 2). Retaining influenza virus infections among both cases and controls reduced VE in 12–64-year-olds by −8% relative to primary (36%; 95% CI: −29 to 68), also then steadily increasing with exclusion of co-infections. With restriction to ILI participants, VE increased by + 9% among 12–64-year-olds, also further increasing with exclusion of co-infections, as illustrated in Supplementary Figure S4.

The VE estimates stratified by WGS findings are shown in Supplementary Figure S5. Restricted to case viruses that failed sequencing, VE was 26% (95%CI: −63 to 66); it increased with restriction to sequenced case viruses at 53% (95%CI: 24 to 71), and in variant-specific analysis for XFG at 63% (95%CI: 30 to 80). Within the subset of failed sequences there were more co-infections (22/74; 30%), notably influenza (16/22; 73%), than among the sequenced subset (13/235; 6%) (p < 0.001) (Supplementary Table S2). Among failed sequences associated with co-infections, Cq values were available for 21/22 with 20/21 showing higher viral load (lower Cq value) for the co-infecting than SARS-CoV-2 virus, with median Cq shift of 10.3 (interquartile range (IQR): 5.1–13.1, range: 1.9–20.6) among the 20 non-SARS-CoV-2 dominant samples. Among sequenced viruses, Cq values were available for 10/13, lower among 3/10 for the co-infecting virus, including Cq shifts of 0.4, 6.0 and 9.8.

## Discussion

The Canadian SPSN reports that, at median 9 weeks post-vaccination, the 2025/26 LP.8.1 vaccine reduced the risk of medically attended outpatient COVID-19 by about half among vaccinated relative to unvaccinated participants, similar if slightly higher among ≥ 65 (53%) than 12–64-year-olds (44%). Our findings uniquely include WGS of contributing case viruses and insights into the potential impact of influenza and other respiratory viruses on VE estimation.

With three-quarters of case viruses genetically characterised, our LP.8.1 VE findings foremost reflect cross-protection against non-LP.8.1 lineages, mostly XFG but also NB.1.8.1. Predominant XFG and NB.1.8.1 variants are distinguished from the 2025/26 vaccine by multiple substitutions affecting the receptor binding and N-terminal domains, including two with potential glycosylation effects [21,22]. Immunogenicity studies nevertheless show LP.8.1 vaccination of immune-experienced people increases cross-reactive neutralising antibody (nAb) against both XFG and NB.1.8.1 [23-25]. With lower XFG nAb titres overall, post-vaccination fold-increases are higher for XFG than NB.1.8.1, reflecting lower XFG relatedness to prior vaccine and circulating strains, but higher XFG relatedness to LP.8.1 [23-25]. Greater breadth of cross-reactivity among older individuals (aged ≥ 65 years), including higher LP.8.1-induced nAb responses against both XFG and NB.1.8.1 [24], aligns with our slightly higher COVID-19 VE among ≥ 65-year-olds. Finally, the LP.8.1 vaccine induces only low titres against the strongly immune-evasive BA.3.2 variant, found sporadically in our dataset through week 09 but showing more recent increase in Ontario and elsewhere, especially among children [26-29].

We explored COVID-19 VE findings in the context of an intense influenza epidemic. In primary analysis, we excluded influenza virus infections from controls to address the potential bias of correlated influenza and COVID-19 vaccination [10]. Retaining influenza virus infections among controls had minimal impact overall but this varied by age, reducing VE relative to primary analysis for 12–64 (−8%) but not ≥ 65-year-olds. While uncertainty given wide CIs must be acknowledged, these divergent effects by age are consistent with the greater influenza contribution, higher influenza VE [13], and stronger association between influenza and COVID-19 vaccination within the younger subset. Excluding RSV infections from controls ≥ 65 years had minimal impact, consistent with their low vaccine coverage (< 25%) and proportion infected (86/875; 10%). As anticipated, the exclusion of other non-vaccine preventable infections from the control series had negligible impact on VE estimates in either age group.

In primary analysis we retained influenza and other respiratory co-infections among COVID-19 cases. Their exclusion in sensitivity analyses was meant to address other considerations such as differential exposure, vulnerability, or severity risks. Given heightened influenza but low-level SARS-CoV-2 circulation, excluding co-infections also addressed non-primary SARS-CoV-2 infections. The impact of excluding co-infections also varied by age, increasing COVID-19 VE estimates among the more often co-infected 12–64 but not ≥ 65-year-olds. Unlike the control series, the additional exclusion of non-vaccine preventable infections from the case series further increased VE estimates but with few vaccinated cases to begin with, uncertainty must again be acknowledged.

A role for co-infections was separately spotlighted in subset restriction based on WGS findings. At face value, higher variant-specific XFG VE compared with overall primary analysis implies much lower VE (greater immune escape) among other contributing case viruses. Instead, we found higher VE more generally among the broader category of sequenced vs non-sequenced case viruses. While emerging, more immune evasive variants (e.g. BA.3.2) may be more likely to fail sequencing coverage thresholds due to limitations in genome assembly pipelines [30], such explanation is highly speculative. Further investigation instead revealed an underlying selection bias favouring higher variant-specific VE. Semi-quantification available for 31/35 co-infections showed most (23/31) had higher viral loads for the co-infecting virus than SARS-CoV-2, with 10 Cq shift. Acknowledging that PCR viral target sensitivities vary, these findings reflect meaningful differences consistent with non-primary SARS-CoV-2 infections. The COVID-19 case viruses that failed WGS were more often associated with co-infections and correspondingly lower viral loads, contributing to overall under-estimation of VE against medical visits that were perhaps with SARS-CoV-2 rather than primarily for COVID-19. Had the co-infecting primary virus been absent, the accompanying SARS-CoV-2 infection alone may not have led to the medical visit as required for eligibility within our COVID-19 case series. We highlight that while the Doll et al. bias is a concern particular to TND VE estimation [10], the potential impact of co-infections would apply to other study designs also.

Our 2025/26 interim COVID-19 VE of 48% among ≥ 12-year-olds is comparable to our previous mid- and end-of-season estimates ranging ca 40–50%, including with restriction to older adults [12,19]. To date just two studies have reported 2025/26 COVID-19 VE, both as TND preprints. A European (5-country) study spanning September to January assessed VE against outpatient COVID-19 among participants ≥ 60 years. With < 20% vaccinated, their analysis included just seven vaccinated cases overall, raising similar sparse data concern as for some of our subset analyses. Primary analysis excluded influenza virus and RSV co-infections from COVID-19 cases but retained influenza virus infections among controls. The VE was 59% (95%CI: 14 to 83) at median 5 weeks post-vaccination [31], comparable to our estimate of 54% for ≥ 65-year-olds under the same conditions but at median 9 weeks. In sensitivity analyses, authors report ≤ 5% change when modifying these conditions, also consistent with our observations among ≥ 65-year-olds. A study from the United States spanning September to November used administrative data to report VE against emergency department, urgent care or outpatient visits for adults ≥ 18 years (half ≥ 65 years), with < 3% vaccinated [32]. Primary analyses retained influenza virus infections and were unadjusted for calendar time (a generally recognised confounder). The VE was 54% (95%CI: 15 to 75) overall against outpatient visits at median 3 weeks post-vaccination, slightly higher than our estimate of 46% among ≥ 12-year-olds at median 9 weeks when similarly retaining influenza virus infections. Excluding influenza virus infections from their controls had no impact, unsurprising given their analysis period largely pre-dated the influenza epidemic, with influenza virus infections comprising < 3% of the control series.

Limitations of the current study include, as elsewhere, low SARS-CoV-2 activity and vaccine coverage, compromising statistical power and precision especially in subset analyses. We note the greatest variability and lowest sample size of vaccinated cases within the 12–64-year age group, warranting cautious interpretation. Despite extensive sensitivity analyses the potential for residual bias and confounding remains. A strength of the SPSN is its systematic and simultaneous testing for multiple respiratory viruses among patients presenting with ARI, but we note 40% still lacked a detectable viral diagnosis based upon the multiplex assays we applied among our participants. Exposure (vaccination) misclassification is unlikely for participants with PIR record of receipt but the default assumption of being unvaccinated in the absence of PIR documentation of LP.8.1 receipt may have under-estimated coverage. Sensitivity analyses excluding participants without any PIR record of COVID-19 vaccination at any time since the pandemic did not alter VE estimates and among participants with known self-reported vaccination status, per cent PIR agreement exceeded 90% overall and by case status, higher for 12–64-year-olds (95%) than ≥ 65-year-olds (86%) (data not shown). More detailed comparison of vaccination agreement metrics and VE estimates are underway. Findings may not generalise to other COVID-19 outcomes, subgroups or settings with a different mix of contributing variants, vaccine target groups, vaccination or infection histories. The time since vaccination underpinning our LP.8.1 VE analyses was relatively short (median: 9 weeks), albeit longer than other available analyses to date for the 2025/26 season (median: 3–5 weeks). The impact of other respiratory virus infections on COVID-19 VE estimation requires check elsewhere and in larger studies, but given effects are expected to vary with proportionate contribution (or relative dilution) within the particular case and/or control series, such check should be routinely undertaken as feasible, notably including subset analyses. In that regard, we note that the time-varying incidence of influenza across the season may also require consideration in period-specific comparisons subset by calendar time or time since vaccination intervals (e.g. in assessing waning protection).

## Conclusions

Interim estimates of 2025/26 LP.8.1 VE from the Canadian SPSN indicate vaccine reduced the risk of outpatient COVID-19 illness by about half among vaccinated relative to unvaccinated individuals, including older adults. Given COVID-19 vaccination is targeted to people at elevated risk of severe outcomes, this upstream risk reduction through vaccination even in the context of mismatched lineages is highly meaningful to individual protection. In the context of low SARS-CoV-2 but high influenza or other respiratory virus incidence, multiplex testing to identify other primary co-infections among cases, or correlated vaccine-preventable infections among controls, may address potential under-estimation of COVID-19 VE.

## Data Availability

Sequencing data for SPSN SARS-CoV-2 viruses meeting provincial and/or national criteria for upload and their submitting and contributing laboratories can be found on GISAID using the Epi Set ID: EPI_SET_260424qa (https://doi.org/10.55876/gis8.260424qa).

## Supplementary Data

659000 Supplementary Material
