# Supplementary material for: Interim 2025/26 LP.8.1 vaccine effectiveness estimates against COVID-19 from the Canadian Sentinel Practitioner Surveillance Network (SPSN): insights into possible impact of influenza and other respiratory virus co-circulation
Source: Euro Surveill. 2026 May 7;31(18):2600331. doi: 10.2807/1560-7917.ES.2026.31.18.2600331 (PMC13235653; doi:10.2807/1560-7917.ES.2026.31.18.2600331)
Supplement: Supplementary Material [file 26-00331_SupplementaryMaterial.pdf]

Supplementary Material

Interim 2025/26 LP.8.1 vaccine effectiveness estimates against COVID-19 from the Canadian Sentinel Practitioner Surveillance Network (SPSN): insights into possible impact of influenza and other respiratory virus co-circulation

As supporting information alongside the above-named article, this supplementary material is hosted by *Eurosurveillance* on behalf of the authors who remain responsible for the accuracy and appropriateness of the content. The same standards for ethics, copyright, attributions and permissions as for the article apply. Supplements are not edited by *Eurosurveillance* and the journal is not responsible for the maintenance of any links or email addresses provided therein.

Supplementary Material - Table of Contents

Canadian Sentinel Practitioner Surveillance Network (SPSN) 2025/26 interim COVID-19 vaccine effectiveness (VE) analyses 26 October 2025 to 07 March 2026 (weeks 44 to 09)

Supplementary Table S1. Respiratory pathogen targets routinely included in NAAT-testing of Canadian Sentinel Practitioner Surveillance Network (SPSN) specimens, by province for the 2025/26 season 3

Supplementary Table S2. Other respiratory pathogens detected among COVID-19 cases and controls 4

Supplementary Table S3. Participant characteristics, including influenza infections among controls 5

Supplementary Table S4. Lineage characterization of contributing COVID-19 case viruses by province 6

Supplementary Figure S1. 2025/26 COVID-19 VE estimates, primary and sensitivity analyses varying other respiratory virus exclusion criteria with time since vaccination details 7

Supplementary Figure S2. 2025/26 COVID-19 VE estimates, primary and sensitivity analyses modifying covariate adjustment 8

Supplementary Figure S3. 2025/26 COVID-19 VE estimates, primary and sensitivity analyses modifying other exclusion criteria 9

Supplementary Figure S4. 2025/26 COVID-19 VE estimates, primary and with restriction to patients presenting with influenza-like illness (ILI) 10

Supplementary Figure S5. 2025/26 COVID-19 VE estimates, primary and stratified by whole genome sequencing findings 11

References 12

Supplementary Table S1. Respiratory pathogen targets routinely included in NAAT-testing of Canadian Sentinel Practitioner Surveillance Network (SPSN) specimens, by province for the 2025/26 season

Target	Province		
	British Columbia (BC)	Ontario	Québec
Influenza A	✓	✓	✓
Influenza B	✓	✓	✓
Seasonal influenza A(H1)			✓
Influenza A(H1N1)pdm09	✓	✓	✓
Influenza A(H3)	✓	✓	✓
Non-influenza respiratory viruses (NIRV)			
Respiratory syncytial virus (RSV) combined	-	✓	-
RSV-A	✓	-	✓
RSV-B	✓	-	✓
Adenovirus	✓	✓	✓
Bocavirus	✓	-	✓
Seasonal coronavirus combined	-	✓	-
Coronavirus 229E	✓	-	✓
Coronavirus NL63	✓	-	✓
Coronavirus OC43	✓	-	✓
Coronavirus HKU1	✓	-	✓
SARS-CoV-2	✓	✓	✓
EV/RV combined	✓	-	✓
Enterovirus (EV)	-	✓	-
Rhinovirus (RV)	-	✓	-
Human metapneumovirus	✓	✓	✓
Parainfluenza combined	-	✓	-
Parainfluenza 1	✓	-	✓
Parainfluenza 2	✓	-	✓
Parainfluenza 3	✓	-	✓
Parainfluenza 4	✓	-	✓
Atypical bacteria			
Mycoplasma pneumoniae	✓	-	✓
Chlamydophila pneumoniae	✓	-	✓
Legionella pneumophila	✓	-	-

NAAT, nucleic acid amplification test; SARS-CoV-2, severe acute respiratory syndrome coronavirus 2

Displayed are the respiratory pathogen targets routinely included in NAAT-testing of respiratory specimens submitted by Canadian SPSN practitioners.

Supplementary Table S2. Other respiratory pathogens detected among COVID-19 cases and controls

COVID-19 case status and age group	Any[a,b,c]	Other respiratory viruses								Atypical bacteria[b,d]		
		Influenza	RSV	EV/RV	Seasonal coronavirus	Para-influenza	HMPV	Adenovirus	Bocavirus[b,d]	M. pneumoniae	C. pneumoniae	L. pneumophila
COVID-19 test-positive cases (overall primary)												
≥12 years (n=310)	35	21	4	6	2	3	0	0	0	0	0	0
12-64 years (n=215)	28	18	3	4	1	3	0	0	0	0	0	0
≥65 years (n=95)	7	3	1	2	1	0	0	0	0	0	0	0
COVID-19 test-positive cases (restricted to successful WGS)												
≥12 years (n=235)	13	5	3	4	1	0	0	0	0	0	0	0
12-64 years (n=159)	11	4	3	3	1	0	0	0	0	0	0	0
≥65 years (n=76)	2	1	0	1	0	0	0	0	0	0	0	0
COVID-19 test-positive cases (restricted to failed WGS)												
≥12 years (n=74)	22	16	1	2	1	3	0	0	0	0	0	0
12-64 years (n=56)	17	14	0	1	0	3	0	0	0	0	0	0
≥65 years (n=18)	5	2	1	1	1	0	0	0	0	0	0	0
COVID-19 test-negative controls (primary analyses excluding influenza infections)												
≥12 years (n=3492)	1406	NA	341	502	250	186	153	24	7	4	9	0
12-64 years (n=2617)	1038	NA	255	371	176	131	121	22	6	4	9	0
≥65 years (n=875)	368	NA	86	131	74	55	32	2	1	0	0	0
COVID-19 test-negative controls (sensitivity analyses including influenza infections)												
≥12 years (n=5110)	3024[e]	1618	349	535	263	194	156	26	9	4	9	0
12-64 years (n=3948)	2369	1331	260	399	187	136	124	24	8	4	9	0
≥65 years (n=1162)	655	287	89	136	76	58	32	2	1	0	0	0

C. pneumoniae, Chlamydophila pneumoniae; EV/RV, entero-/rhinoviruses; HMPV, human metapneumovirus; L. pneumophila, Legionella pneumophila; M. pneumoniae, Mycoplasma pneumoniae; NA, not applicable; RSV, respiratory syncytial virus; WGS, whole genome sequencing

[a] Owing to co-infections among other respiratory viruses (e.g., one influenza plus EV/RV co-infection among COVID-19 test-positive cases 12-64 years), the "Any" tallies are lower than the sum of individual respiratory virus detections.
[b] The "Any" tallies are meant to reflect specimens excluded from sensitivity analyses. Because bocavirus, M. pneumoniae, C. pneumoniae and L. pneumophila were not tested in Ontario and are retained throughout, they are not captured in the "Any" column tallies. Overall (including influenza infections among controls), if bocavirus and atypical bacteria are also included in the "any" detected infection tallies, there were 3041 respiratory pathogen detections among COVID-19 test-negative controls.
[c] Control specimens with indeterminate (IND) results for any non-SARS-CoV-2 viral targets shown in **Supplementary Table S1** retained throughout (n=4, all targets IND; n=10, influenza target IND; n=4, IND target in a specimen positive for another viral target).
[d] Assessed in British Columbia and Quebec only, not Ontario.
[e] If bocavirus and atypical bacteria are also included in the "any" tallies, there were 3041 COVID-19 controls that tested positive for at least one respiratory pathogen overall.

Supplementary Table S3. Participant characteristics, including influenza infections among controls, Canadian Sentinel Practitioner Surveillance Network (SPSN), 26 October 2025 to 07 March 2026 (weeks 44 – 09) (n =5420)

Characteristics		All ARI participants[a] (column %, unless otherwise specified)						COVID-19 vaccinated, PIR-based[b,c] (row %, unless otherwise specified)					
		Overall		COVID-19 cases		COVID-19 controls		Overall		COVID-19 cases		COVID-19 controls	
		n	%	n	%	n	%	n	%	n	%	n	%
N (row %)		5420	100	310	6	5110	94	765	14	29	9	736	14
Age group (years)													
12-49		3054	56	157	51	2897	57	174	6	5	3	169	6
50-64		1109	20	58	19	1051	21	144	13	4	NC	140	13
65-79		972	18	83	27	889	17	321	33	15	18	306	34
≥80		285	5	12	4	273	5	126	44	5	NC	121	44
Median (IQR)		45 (30-63)		48.5 (34-68)		45 (30-63)		68 (52-76)		72 (63-77)		67 (52-76)	
Sex													
Female		3437	63	210	68	3227	63	481	14	18	9	463	14
Male		1963	36	99	32	1864	36	283	14	11	11	272	15
Unknown		20	0	1	0	19	0	1	NC	0	NC	1	NC
Comorbidity[d]													
No		3635	67	200	65	3435	67	409	11	17	9	392	11
Yes		1327	24	79	25	1248	24	305	23	9	11	296	24
Unknown		458	8	31	10	427	8	51	11	3	NC	48	11
Province													
British Columbia		1160	21	28	9	1132	22	255	22	4	NC	251	22
Ontario		3026	56	222	72	2804	55	343	11	14	6	329	12
Quebec		1234	23	60	19	1174	23	167	14	11	18	156	13
Period of specimen collection (epidemiological week span), 2025/26[e]													
44-47		770	14	56	18	714	14	37	5	2	NC	35	5
48-01		2854	53	109	35	2745	54	412	14	14	13	398	14
02-09		1796	33	145	47	1651	32	316	18	13	9	303	18
COVID-19 spring 2025 vaccination status (PIR-based; column %)[f]													
12-64 years	**Total N**	**4163**	**%**	**215**	**%**	**3948**	**%**	**318**	**%**	**9**	**%**	**309**	**%**
	Yes	15	<1	0	0	15	<1	9	3	0	NC	9	3
	No	4148	100	215	100	3933	100	309	97	9	NC	300	97
≥65 years	**Total N**	**1257**	**%**	**95**	**%**	**1162**	**%**	**447**	**%**	**20**	**%**	**427**	**%**
	Yes	143	11	7	7	136	12	97	22	4	NC	93	22
	No	1114	89	88	93	1026	88	350	78	16	NC	334	78
Seasonal 2025/2026 influenza vaccination status (sentinel-based; column %)[g]													
12-64 years[h]	**Total N**	**4163**	**%**	**215**	**%**	**3948**	**%**	**318**	**%**	**9**	**%**	**309**	**%**
	Yes	809	19	35	16	774	20	275	86	8	NC	267	86
	No	3040	73	159	74	2881	73	26	8	1	NC	25	8
	Unknown	314	8	21	10	293	7	17	5	0	NC	17	6
≥65 years[i]	**Total N**	**1257**	**%**	**95**	**%**	**1162**	**%**	**447**	**%**	**20**	**%**	**427**	**%**
	Yes	657	52	46	48	611	53	358	80	14	NC	344	81
	No	500	40	39	41	461	40	55	12	4	NC	51	12
	Unknown	100	8	10	11	90	8	34	8	2	NC	32	7

ARI, acute respiratory illness; CI, confidence interval; IQR, interquartile range; NC, not calculated (denominator <60); OR, odds ratio; PIR, provincial immunization registry; VE, vaccine effectiveness

[a] Participants as per primary VE analyses: age ≥12 years from SPSN provinces of British Columbia, Ontario and Quebec; documented PIR-based COVID-19 vaccine status ≥2 weeks before illness onset but here including influenza test-positive cases among COVID-19 controls.

[b] Vaccination status PIR-based and refers to LP.8.1 vaccination as part of autumn 2025/26 COVID-19 immunization campaign, received ≥2 weeks before illness onset. Participants without PIR documented record of having received the specified vaccine are considered unvaccinated; those vaccinated <2 weeks are excluded (n=81). Only specimens confirmed by the submitting sentinel to have been collected within 7 days of ARI onset are included. Accordingly, where ARI onset date is missing, specimens collected at least 21 days after vaccination date are considered to have been vaccinated ≥2 weeks before ARI onset (n=9). Conversely, if ARI onset date is missing and the interval between vaccination and specimen collection date is <2 weeks then vaccination assigned as <2 weeks before onset (n=3).

[c] Based on PIR data, earliest LP.8.1 vaccination (any timing or ≥2 weeks before illness onset) was week 39 in Ontario, and week 41 in British Columbia and Quebec. Overall whether based on any timing or ≥2 weeks before onset, >90% were vaccinated by the end of week 48.

[d] Includes chronic comorbidities increasing risk of serious influenza complications as per Canada's National Advisory Committee on Immunization [1]

[e] Missing specimen collection dates were imputed as the date the specimen was received and processed at the laboratory minus 2 days. In VE analyses, calendar time adjustment was undertaken bi-weekly (except weeks 07 to 09).

[f] Defined by participants (cases or controls) KP.2 vaccinated between 01 April 2025 and 30 September 2025 (BC and Quebec) or 01 April 2025 and 17 September 2025 (Ontario), as per PIR documentation. Last PIR-recorded date of such receipt was June 2025 in British Columbia, July 2025 in Ontario, and August 2025 in Quebec.

[g] Sentinel-based report of 2025/26 seasonal influenza vaccination without regard to timing in relation to illness onset.

[h] OR, influenza vaccination (yes vs no or unknown) for COVID-19 vaccinated (267/309; 86%) vs unvaccinated (507/3639; 14%) controls: 39.3 (95% CI: 28.0 to 55.1), similar but more pronounced when excluding those with unknown self-reported influenza vaccination status.

[i] OR, influenza vaccination (yes vs no or unknown) for COVID-19 vaccinated (344/427; 81%) vs unvaccinated (267/735; 36%) controls: 7.3 (95% CI: 5.5 to 9.6), similar but more pronounced when excluding those with unknown self-reported influenza vaccination status.

Supplementary Table S4. Lineage characterization of contributing COVID-19 case viruses by province, Canadian Sentinel Practitioner Surveillance Network (SPSN), 26 October 2025 to 07 March 2026 (weeks 44 - 09) (n = 310)

Case viruses			BC<br>N = 28	Ontario<br>N = 222	Quebec<br>N = 60	TOTAL<br>N = 310
Case viruses successfully sequenced, n (%, n/N)			24 (86%)	157 (71%)	54 (90%)	235 (76%)
Parental lineage[a]	Sub-lineages detected among SPSN case viruses[a,b]	# of spike protein mutations relative to LP.8.1 vaccine[c]				
NB.1.8.1	PQ.*	10[d]	3 (NC)	31 (20%)	12 (NC)	46 (20%)
XFG	QF.*, QK.2, QS.2, RM.1, RN.*, RV.1	8[e]	19 (NC)	107 (68%)	38 (NC)	164 (70%)
BA.3.2	RE.*	64[f]		4 (3%)		4 (2%)
OTHER	LF.7.9.1.*, MC.10.*, XFQ, XFV, XFY.*, XFZ.*, XGN	NA	2 (NC)	15 (10%)	4 (NC)	21 (9%)

BC, British Columbia; NA, not applicable; NC, not calculated (denominator <60); NTD, N-terminal domain; RBD, receptor-binding domain; +/-CHO, potential gain/loss of glycosylation

Whole genome sequencing of SPSN SARS-CoV-2 case viruses followed routine provincial or national laboratory protocols [5–10] and lineages assigned based on contemporary Pango nomenclature [2,11]. Data for SPSN SARS-CoV-2 viruses meeting provincial and/or national criteria for upload and their submitting and contributing laboratories can be found on GISAID using the Epi Set ID:EPI_SET_260424qa (https://doi.org/10.55876/gis8.260424qa) [12].

[a] Lineages determined using pangolin version 4.3.1, data version v1.38 [2]

[b] Lineages suffixed with an asterisk also include their descendant lineages, unless otherwise noted

[c] Parental lineage-defining mutations defined as per pango-lineage designations (https://github.com/cov-lineages/pango-designation/tree/master) updated March 25, 2026

[d] T22N (NTD)(+CHO), S31ins (NTD)(-CHO), F59S (NTD), R182K (NTD), G184S (NTD), L186F (NTD), T346R (RBD), A435S (RBD), R445H (RBD), K478I (RBD)

[e] T22N (NTD)(+CHO), P31ins (NTD)(-CHO), R182K (NTD), L186F (NTD), S190R (NTD), K444R (RBD), N487D (RBD), T572I (S1)

[f] I19T (NTD), L24ins (NTD), P25ins (NTD), L26ins (NTD), S27A (NTD), S31ins (NTD), L50S (NTD), A67V (NTD), T95I (NTD), I101T (NTD), F127V (NTD), 136-147del (NTD), G158R (NTD), N164K (NTD), S172F (NTD), L186F (NTD), K187T (NTD), S190R (NTD), G213V (NTD), F216L (NTD), 243-244del (NTD), N245H (NTD), P251S (NTD), D264A (NTD), V332I (RBD), H339Y (RBD), T346R (RBD), A348P (RBD), A376T (RBD), A435S (RBD), K440R (RBD), R445A (RBD), S446D (RBD), D450N (RBD), S455L (RBD), L456F (RBD), K478N (RBD), K481N (RBD), V483ins (RBD), P486F (RBD), E493Q (RBD), G496S (RBD), H505Y(RBD), K529N (S1), K554D (S1), V570A (S1), E583D (S1), S621P (S1), H625R (S1), N641K (S1), V642G (S1), E654K (S1), K679R (S1) [3,4]

Supplementary Figure S1. 2025/26 COVID-19 VE estimates, primary and sensitivity analyses varying other respiratory virus exclusion criteria, overall and by age group with time since vaccination details, Canadian Sentinel Practitioner Surveillance Network (SPSN), 26 October 2025 to 07 March 2026 (weeks 44 to 09) (n =3802)

	Cases, n		Controls, n			Cases, TSV (weeks)		Controls, TSV (weeks)	
	Vac (%)	Total	Vac (%)	Total	VE (95% CI)	Median (IQR)	Range	Median (IQR)	Range
≥12 years									
**Excl. influenza infections from controls**	**29 (9%)**	**310**	**577 (17%)**	**3492**	**48 (21, 66)**	**7 (4−13)**	**2−19**	**9 (5−13)**	**2−21**
Excl. influenza and RSV infections from controls	29 (9%)	310	502 (16%)	3151	46 (17, 64)	7 (4−13)	2−19	9 (5−13)	2−21
Excl. all known viral infections from controls	29 (9%)	310	315 (15%)	2086	44 (15, 64)	7 (4−13)	2−19	9 (5−12)	2−20
Excl. influenza co−infections from cases	27 (9%)	289	577 (17%)	3492	50 (24, 68)	7 (5−13)	2−19	9 (5−13)	2−21
Excl. influenza and RSV co−infections from cases	25 (9%)	285	577 (17%)	3492	54 (28, 71)	7 (5−13)	2−19	9 (5−13)	2−21
Excl. all known viral co−infections from cases	23 (8%)	275	577 (17%)	3492	57 (31, 73)	7 (5−14)	2−19	9 (5−13)	2−21
Incl. influenza infections in controls	29 (9%)	310	736 (14%)	5110	46 (17, 64)	7 (4−13)	2−19	8 (5−12)	2−21
Excl. influenza co−infections from cases	27 (9%)	289	736 (14%)	5110	48 (20, 66)	7 (5−13)	2−19	8 (5−12)	2−21
Excl. influenza and RSV co−infections from cases	25 (9%)	285	736 (14%)	5110	52 (25, 69)	7 (5−13)	2−19	8 (5−12)	2−21
Excl. all known viral co−infections from cases	23 (8%)	275	736 (14%)	5110	55 (28, 72)	7 (5−14)	2−19	8 (5−12)	2−21
12−64 years									
**Excl. influenza infections from controls**	**9 (4%)**	**215**	**247 (9%)**	**2617**	**44 (−12, 72)**	**7 (3−7)**	**3−16**	**9 (5−13)**	**2−19**
Excl. influenza and RSV infections from controls	9 (4%)	215	211 (9%)	2362	40 (−20, 70)	7 (3−7)	3−16	9 (5−14)	2−19
Excl. all known viral infections from controls	9 (4%)	215	134 (8%)	1579	43 (−17, 72)	7 (3−7)	3−16	9 (5−14)	2−19
Excl. influenza co−infections from cases	7 (4%)	197	247 (9%)	2617	55 (1, 79)	7 (3−13)	3−16	9 (5−13)	2−19
Excl. influenza and RSV co−infections from cases	6 (3%)	194	247 (9%)	2617	61 (9, 83)	7 (3−13)	3−16	9 (5−13)	2−19
Excl. all known viral co−infections from cases	5 (3%)	187	247 (9%)	2617	66 (15, 86)	7 (3−7)	3−16	9 (5−13)	2−19
Incl. influenza infections in controls	9 (4%)	215	309 (8%)	3948	36 (−29, 68)	7 (3−7)	3−16	8 (5−12)	2−19
Excl. influenza co−infections from cases	7 (4%)	197	309 (8%)	3948	48 (−12, 76)	7 (3−13)	3−16	8 (5−12)	2−19
Excl. influenza and RSV co−infections from cases	6 (3%)	194	309 (8%)	3948	55 (−3, 81)	7 (3−13)	3−16	8 (5−12)	2−19
Excl. all known viral co−infections from cases	5 (3%)	187	309 (8%)	3948	61 (3, 84)	7 (3−7)	3−16	8 (5−12)	2−19
≥65 years									
**Excl. influenza infections from controls**	**20 (21%)**	**95**	**330 (38%)**	**875**	**53 (21, 73)**	**7.5 (5−13.5)**	**2−19**	**9 (5−13)**	**2−21**
Excl. influenza and RSV infections from controls	20 (21%)	95	291 (37%)	789	53 (20, 73)	7.5 (5−13.5)	2−19	9 (5−12)	2−21
Excl. all known viral infections from controls	20 (21%)	95	181 (36%)	507	50 (13, 71)	7.5 (5−13.5)	2−19	9 (5−12)	2−20
Excl. influenza co−infections from cases	20 (22%)	92	330 (38%)	875	51 (17, 72)	7.5 (5−13.5)	2−19	9 (5−13)	2−21
Excl. influenza and RSV co−infections from cases	19 (21%)	91	330 (38%)	875	54 (21, 73)	8 (5−14)	2−19	9 (5−13)	2−21
Excl. all known viral co−infections from cases	18 (20%)	88	330 (38%)	875	55 (22, 74)	8.5 (5−14)	2−19	9 (5−13)	2−21
Incl. influenza infections in controls	20 (21%)	95	427 (37%)	1162	53 (21, 72)	7.5 (5−13.5)	2−19	8 (5−11)	2−21
Excl. influenza co−infections from cases	20 (22%)	92	427 (37%)	1162	51 (17, 71)	7.5 (5−13.5)	2−19	8 (5−11)	2−21
Excl. influenza and RSV co−infections from cases	19 (21%)	91	427 (37%)	1162	54 (21, 73)	8 (5−14)	2−19	8 (5−11)	2−21
Excl. all known viral co−infections from cases	18 (20%)	88	427 (37%)	1162	55 (22, 74)	8.5 (5−14)	2−19	8 (5−11)	2−21

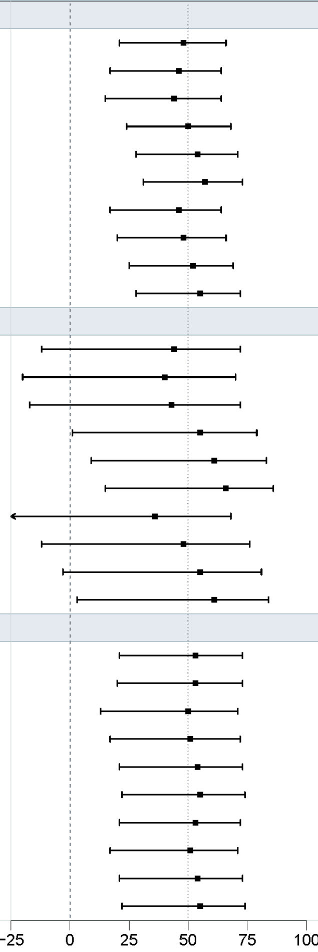

CI, confidence interval; Excl., excluding; Incl., including; IQR, interquartile range; TSV, time since vaccination; Vac, vaccinated; VE, vaccine effectiveness

Primary VE analyses are shown in bold. Primary analyses are without regard to prior COVID-19 vaccination or infection history (including 2025 spring vaccinated) and exclude influenza infections from COVID-19 controls (retaining influenza co-infections among COVID-19 cases). Sensitivity analyses explore VE by modifying specified inclusion or exclusion criteria related to influenza, RSV and other respiratory virus infections among COVID-19 cases and/or controls. Analyses are restricted to participants ≥12 years from the provinces of British Columbia (BC), Ontario or Quebec. Current vaccination status is based upon provincial immunization registry documentation of 2025/26 LP.8.1 COVID-19 vaccine receipt. VE is assessed at ≥2 weeks post-vaccination; participants vaccinated <2 weeks before illness onset excluded. All VE estimates are adjusted for age group (12-49, 50-64, ≥65 years), province (BC, Ontario, Quebec), and calendar time (based upon specimen collection date, bi-weekly except weeks 07 to 09). TSV refers to interval between 2025/26 COVID-19 vaccination and acute respiratory illness onset among vaccinated participants, except those for whom onset date is unknown, assigned instead based on specimen collection date (n=9). Control specimens with indeterminate (IND) results for any non-SARS-CoV-2 viral targets and those positive for bocavirus or atypical bacteria (because not assessed in Ontario) are retained throughout (**Supplementary Tables S1 and S2**).

Supplementary Figure S2. 2025/26 COVID-19 VE estimates, primary and sensitivity analyses modifying covariate adjustment, overall and by age group, Canadian Sentinel Practitioner Surveillance Network (SPSN), 26 October 2025 to 07 March 2026 (weeks 44 to 09) (n =3802)

	Cases, n		Controls, n			Cases, TSV (weeks)		Controls, TSV (weeks)	
	Vac (%)	Total	Vac (%)	Total	VE (95% CI)	Median (IQR)	Range	Median (IQR)	Range
≥12 years									
**Adjusted for age, province, calendar time**	**29 (9%)**	**310**	**577 (17%)**	**3492**	**48 (21, 66)**	**7 (4−13)**	**2−19**	**9 (5−13)**	**2−21**
Additional adjustment for sex	29 (9%)	309	576 (17%)	3481	48 (21, 66)	7 (4−13)	2−19	9 (5−13)	2−21
Additional adjustment for comorbidity	26 (9%)	279	532 (17%)	3180	47 (18, 66)	7 (3−13)	2−19	9 (5−13)	2−21
Additional adjustment for sex and comorbidity	26 (9%)	278	532 (17%)	3172	47 (18, 66)	7 (3−13)	2−19	9 (5−13)	2−21
Additional adjustment for finer age strata	29 (9%)	310	577 (17%)	3492	47 (19, 65)	7 (4−13)	2−19	9 (5−13)	2−21
12−64 years									
**Adjusted for age, province, calendar time**	**9 (4%)**	**215**	**247 (9%)**	**2617**	**44 (−12, 72)**	**7 (3−7)**	**3−16**	**9 (5−13)**	**2−19**
Additional adjustment for sex	9 (4%)	214	246 (9%)	2606	44 (−12, 72)	7 (3−7)	3−16	9 (5−13)	2−19
Additional adjustment for comorbidity	8 (4%)	197	234 (10%)	2400	47 (−11, 74)	5.5 (3−7)	3−16	9 (5−13)	2−19
Additional adjustment for sex and comorbidity	8 (4%)	196	234 (10%)	2392	46 (−12, 74)	5.5 (3−7)	3−16	9 (5−13)	2−19
Additional adjustment for finer age strata	9 (4%)	215	247 (9%)	2617	45 (−11, 72)	7 (3−7)	3−16	9 (5−13)	2−19
≥65 years									
**Adjusted for age, province, calendar time**	**20 (21%)**	**95**	**330 (38%)**	**875**	**53 (21, 73)**	**7.5 (5−13.5)**	**2−19**	**9 (5−13)**	**2−21**
Additional adjustment for sex	20 (21%)	95	330 (38%)	875	53 (21, 73)	7.5 (5−13.5)	2−19	9 (5−13)	2−21
Additional adjustment for comorbidity	18 (22%)	82	298 (38%)	780	51 (14, 72)	8.5 (5−14)	2−19	9 (5−12)	2−21
Additional adjustment for sex and comorbidity	18 (22%)	82	298 (38%)	780	51 (14, 72)	8.5 (5−14)	2−19	9 (5−12)	2−21
Additional adjustment for finer age strata	20 (21%)	95	330 (38%)	875	51 (17, 71)	7.5 (5−13.5)	2−19	9 (5−13)	2−21

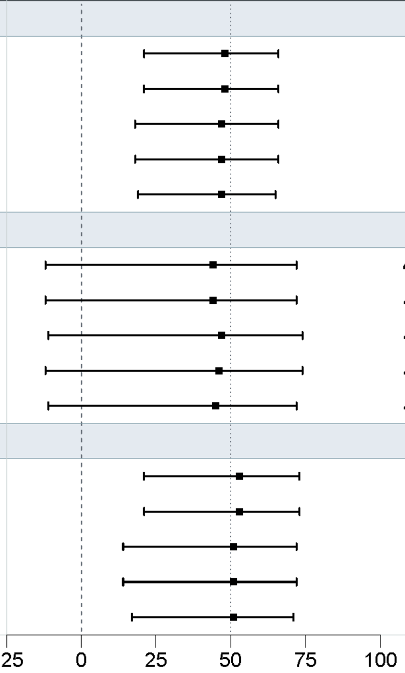

CI, confidence interval; Excl., excluding; Incl., including; IQR, interquartile range; TSV, time since vaccination; Vac, vaccinated; VE, vaccine effectiveness

Primary VE analyses are shown in bold. Primary analyses are adjusted for age group (12-49, 50-64, >65 years), province (British Columbia (BC), Ontario, Quebec), and calendar time (based upon specimen collection date, bi-weekly except weeks 07 to 09). Sensitivity analyses explore VE with additional adjustment for sex (male/female), comorbidity (yes/no), or both, excluding participants with missing information for the specified covariate(s); and adjusting for finer age strata (12-24, 25-49, 50-64, 65-79, >80 years).

Current vaccination status based upon provincial immunization registry documentation of 2025/26 LP.8.1 COVID-19 vaccine receipt. Analyses restricted to participants >12 years from the provinces of BC, Ontario or Quebec. VE assessed at >2 weeks post-vaccination; participants vaccinated <2 weeks before illness onset excluded. VE analyses are without regard to prior COVID-19 vaccination history and exclude influenza infections from COVID-19 controls (retaining influenza co-infections among COVID-19 cases). TSV refers to interval between 2025/26 COVID-19 vaccination and acute respiratory illness onset among vaccinated participants, except those for whom onset date is unknown, assigned instead based on specimen collection date (n=9).

Supplementary Figure S3. 2025/26 COVID-19 VE estimates, primary and sensitivity analyses modifying other exclusion criteria, overall and by age group, Canadian Sentinel Practitioner Surveillance Network (SPSN), 26 October 2025 to 07 March 2026 (weeks 44 to 09) (n =3802)

	Cases, n		Controls, n			Cases, TSV (weeks)		Controls, TSV (weeks)	
	Vac (%)	Total	Vac (%)	Total	VE (95% CI)	Median (IQR)	Range	Median (IQR)	Range
≥12 years									
**Incl. 2025 spring vaccinated, incl. participants without PIR record**	**29 (9%)**	**310**	**577 (17%)**	**3492**	**48 (21, 66)**	**7 (4−13)**	**2−19**	**9 (5−13)**	**2−21**
Excl. 2025 spring vaccinated	25 (8%)	303	496 (15%)	3372	49 (20, 67)	7 (3−11)	2−19	9 (5−13)	2−21
Excl. participants without PIR record	29 (10%)	277	577 (18%)	3192	48 (21, 66)	7 (4−13)	2−19	9 (5−13)	2−21
12−64 years									
**Incl. 2025 spring vaccinated, incl. participants without PIR record**	**9 (4%)**	**215**	**247 (9%)**	**2617**	**44 (−12, 72)**	**7 (3−7)**	**3−16**	**9 (5−13)**	**2−19**
Excl. 2025 spring vaccinated	9 (4%)	215	238 (9%)	2604	42 (−15, 71)	7 (3−7)	3−16	9 (5−13)	2−19
Excl. participants without PIR record	9 (5%)	185	247 (11%)	2343	42 (−16, 71)	7 (3−7)	3−16	9 (5−13)	2−19
≥65 years									
**Incl. 2025 spring vaccinated, incl. participants without PIR record**	**20 (21%)**	**95**	**330 (38%)**	**875**	**53 (21, 73)**	**7.5 (5−13.5)**	**2−19**	**9 (5−13)**	**2−21**
Excl. 2025 spring vaccinated	16 (18%)	88	258 (34%)	768	55 (20, 75)	7 (4−12)	2−19	9 (5−12)	2−21
Excl. participants without PIR record	20 (22%)	92	330 (39%)	849	54 (22, 73)	7.5 (5−13.5)	2−19	9 (5−13)	2−21

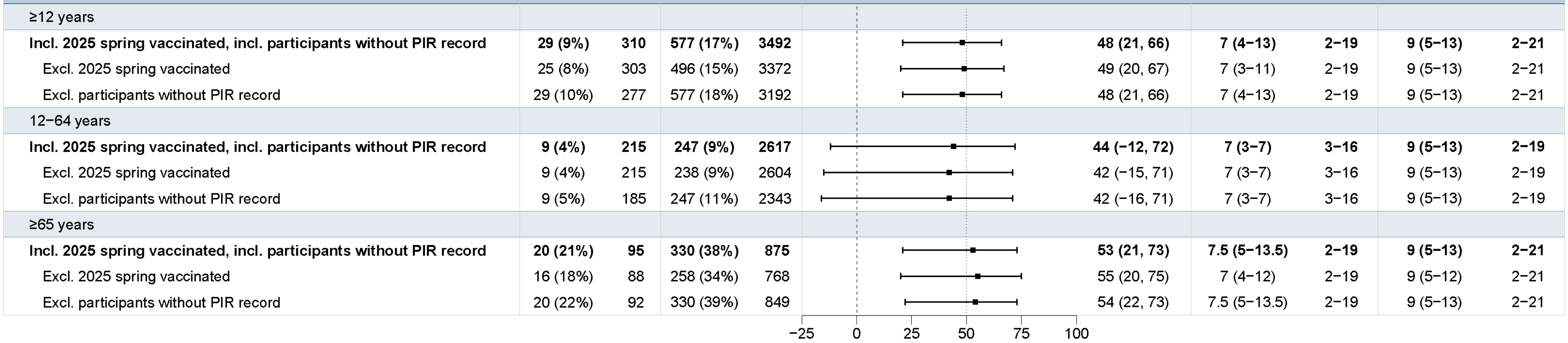

CI, confidence interval; Excl., excluding; Incl., including; IQR, interquartile range; PIR, provincial immunization registry; TSV, time since vaccination; Vac, vaccinated; VE, vaccine

Primary VE analyses are shown in bold. Primary analyses are without regard to prior COVID-19 vaccination history (including 2025 spring vaccinated) and exclude influenza infections from COVID-19 controls (retaining influenza co-infections among COVID-19 cases). Sensitivity analyses explore VE by modifying specified inclusion or exclusion criteria, namely: excluding 2025 spring vaccinated; and excluding participants who had no identified doses or record within the COVID-19 PIR at any time (n=333) (rather than default assignment as unvaccinated). The 2025 spring vaccinated are defined by PIR-documented receipt of KP.2 vaccine between 01 April 2025 and 30 September 2025 (BC or Quebec) or 01 April 2025 and 17 September 2025 (Ontario). Based on PIR documentation, last date of such receipt was June in BC, July in Ontario, and August in Quebec.

Current vaccination status based upon PIR documentation of 2025/26 LP.8.1 COVID-19 vaccine receipt. Analyses restricted to participants ≥12 years from the provinces of British Columbia (BC), Ontario or Quebec. VE assessed at ≥2 weeks post-vaccination; participants vaccinated <2 weeks before illness onset excluded. All estimates are adjusted for age group (12-49, 50-64, ≥65 years), province (BC, Ontario, Quebec), and calendar time (based upon specimen collection date, bi-weekly except weeks 07 to 09). TSV refers to interval between 2025/26 COVID-19 vaccination and acute respiratory illness onset among vaccinated participants, except those for whom onset date is unknown, assigned instead based on specimen collection date (n=9).

Supplementary Figure S4. 2025/26 COVID-19 VE estimates, primary and with restriction to patients presenting with influenza-like illness (ILI), overall and by age group, Canadian Sentinel Practitioner Surveillance Network (SPSN), 26 October 2025 to 07 March 2026 (weeks 44 to 09) (n =3802)

	Cases, n		Controls, n			Cases, TSV (weeks)		Controls, TSV (weeks)	
	Vac (%)	Total	Vac (%)	Total	VE (95% CI)	Median (IQR)	Range	Median (IQR)	Range
≥12 years									
**Acute respiratory illness (ARI)**	**29 (9%)**	**310**	**577 (17%)**	**3492**	**48 (21, 66)**	**7 (4−13)**	**2−19**	**9 (5−13)**	**2−21**
Influenza−like illness (ILI)	22 (8%)	265	450 (16%)	2754	52 (23, 70)	7 (3−13)	2−18	9 (5−13)	2−20
Excl. influenza co−infections from cases	20 (8%)	246	450 (16%)	2754	56 (28, 73)	7 (4−13)	2−18	9 (5−13)	2−20
Excl. influenza and RSV co−infections from cases	19 (8%)	243	450 (16%)	2754	58 (30, 74)	7 (3−13)	2−18	9 (5−13)	2−20
Excl. all known viral co−infections from cases	17 (7%)	235	450 (16%)	2754	61 (34, 77)	7 (5−13)	2−18	9 (5−13)	2−20
12−64 years									
**Acute respiratory illness (ARI)**	**9 (4%)**	**215**	**247 (9%)**	**2617**	**44 (−12, 72)**	**7 (3−7)**	**3−16**	**9 (5−13)**	**2−19**
Influenza−like illness (ILI)	7 (4%)	188	202 (10%)	2054	53 (−4, 78)	7 (3−7)	3−16	9 (5−13)	2−19
Excl. influenza co−infections from cases	5 (3%)	171	202 (10%)	2054	65 (11, 86)	7 (7−7)	3−16	9 (5−13)	2−19
Excl. influenza and RSV co−infections from cases	5 (3%)	169	202 (10%)	2054	64 (9, 86)	7 (7−7)	3−16	9 (5−13)	2−19
Excl. all known viral co−infections from cases	4 (2%)	163	202 (10%)	2054	70 (17, 89)	7 (5−11.5)	3−16	9 (5−13)	2−19
≥65 years									
**Acute respiratory illness (ARI)**	**20 (21%)**	**95**	**330 (38%)**	**875**	**53 (21, 73)**	**7.5 (5−13.5)**	**2−19**	**9 (5−13)**	**2−21**
Influenza−like illness (ILI)	15 (19%)	77	248 (35%)	700	54 (17, 75)	7 (3−13)	2−18	9 (5.5−13)	2−20
Excl. influenza co−infections from cases	15 (20%)	75	248 (35%)	700	53 (14, 74)	7 (3−13)	2−18	9 (5.5−13)	2−20
Excl. influenza and RSV co−infections from cases	14 (19%)	74	248 (35%)	700	57 (19, 77)	7.5 (3−13)	2−18	9 (5.5−13)	2−20
Excl. all known viral co−infections from cases	13 (18%)	72	248 (35%)	700	59 (22, 78)	9 (5−13)	2−18	9 (5.5−13)	2−20

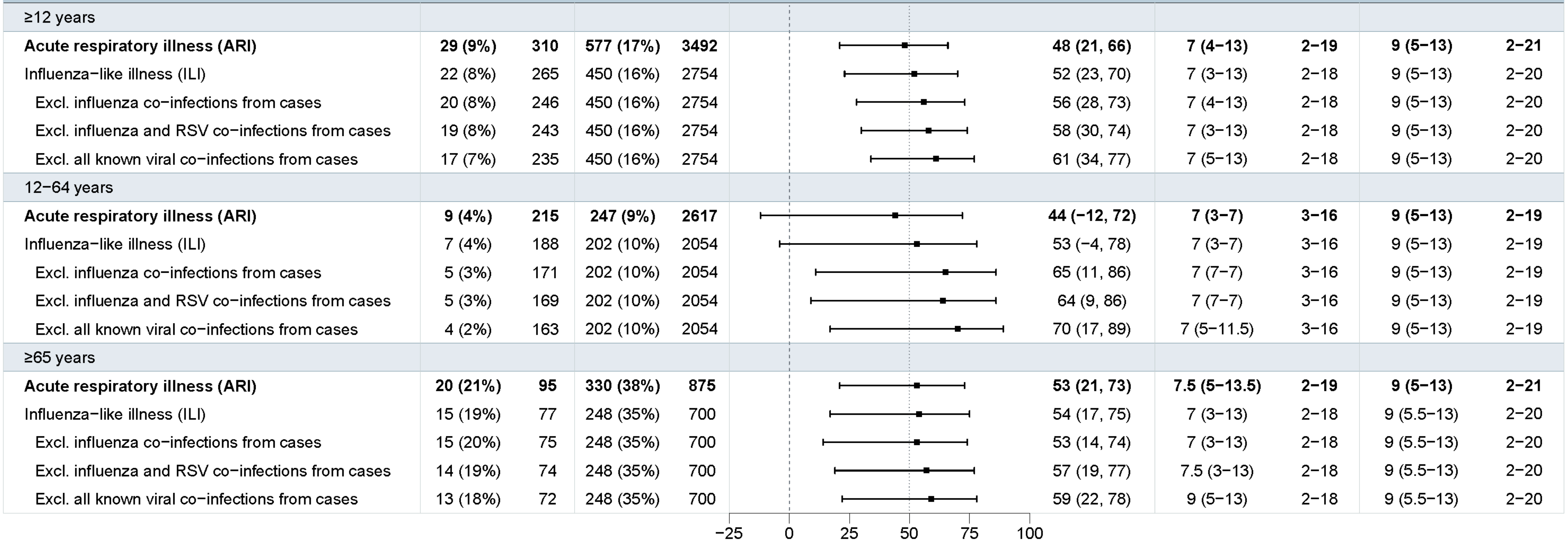

CI, confidence interval; Excl., excluding; IQR, interquartile range; TSV, time since vaccination; Vac, vaccinated; VE, vaccine

Primary VE analyses are shown in bold. Primary analyses include patients with ARI (defined by new or worsening cough that could be due to a respiratory infection). Sensitivity analyses explore VE restricted to participants with ILI defined by fever (or feverishness or chills) and cough plus one or more of: sore throat, myalgia, arthralgia or prostration. Patients ≥65 years do not require fever to meet the ILI case definition.

Current vaccination status based upon provincial immunization registry documentation of 2025/26 LP.8.1 COVID-19 vaccine receipt. Analyses restricted to participants ≥12 years from the provinces of British Columbia (BC), Ontario or Quebec. VE assessed at ≥2 weeks post-vaccination; participants vaccinated <2 weeks before illness onset excluded. VE analyses are without regard to prior COVID-19 vaccination history and exclude influenza infections from COVID-19 controls (retaining influenza co-infections among COVID-19 cases). All estimates are adjusted for age group (12-49, 50-64, ≥65 years), province (BC, Ontario, Quebec), and calendar time (based upon specimen collection date, bi-weekly except weeks 07 to 09). TSV refers to interval between 2025/26 COVID-19 vaccination and acute respiratory illness onset among vaccinated participants, except those for whom onset date is unknown, assigned instead based on specimen collection date (n=9).

Supplementary Figure S5. 2025/26 COVID-19 VE estimates, primary and stratified by whole genome sequencing findings, overall participants, Canadian Sentinel Practitioner Surveillance Network (SPSN), 26 October 2025 to 07 March 2026 (weeks 44 to 09) (n =3802)

	Cases, n		Controls, n			Cases, TSV (weeks)		Controls, TSV (weeks)	
	Vac (%)	Total	Vac (%)	Total	VE (95% CI)	Median (IQR)	Range	Median (IQR)	Range
**All case viruses**	**29 (9%)**	**310**	**577 (17%)**	**3492**	**48 (21, 66)**	**7 (4−13)**	**2−19**	**9 (5−13)**	**2−21**
Case viruses successfully characterized by WGS	21 (9%)	235	577 (17%)	3492	53 (24, 71)	7 (5−14)	2−19	9 (5−13)	2−21
XFG−lineage case viruses	12 (7%)	164	577 (17%)	3492	63 (30, 80)	8 (5−13)	2−16	9 (5−13)	2−21
Case viruses not successfully characterized by WGS	8 (11%)	74	577 (17%)	3492	26 (−63, 66)	6.5 (3.5−7.5)	3−13	9 (5−13)	2−21

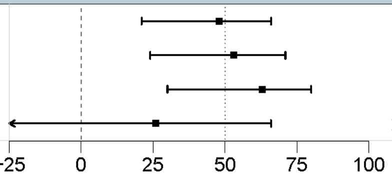

CI, confidence interval; IQR, interquartile range; TSV, time since vaccination; Vac, vaccinated; VE, vaccine effectiveness; WGS, whole genome sequencing

Primary VE analyses among participants ≥12 years of age are shown in bold. Primary analyses include all contributing COVID-19 case viruses. Displayed subset analyses explore VE with restriction based upon WGS, namely: restriction to case viruses that were (n=235) or were not (n=74) successfully characterized by WGS, excluding 1 case virus for which WGS remains pending. Among case viruses successfully sequenced by WGS, VE analyses were further limited to the subset identified as XFG-lineage case viruses (Pango nomenclature) [2]. Case viruses not successfully characterized by WGS include those failed sequencing (n = 29) as well as those not sequenced due to cycle threshold ≥30 (n=45, all from Ontario).

Current vaccination status based upon provincial immunization registry documentation of 2025/26 LP.8.1 COVID-19 vaccine receipt. Analyses restricted to participants ≥12 years from the provinces of British Columbia (BC), Ontario or Quebec. VE assessed at ≥2 weeks post-vaccination; participants vaccinated <2 weeks before illness onset excluded. VE analyses are without regard to prior COVID-19 vaccination history and exclude influenza infections from COVID-19 controls (retaining influenza co-infections among COVID-19 cases). All estimates are adjusted for age group (12-49, 50-64, ≥65 years), province (BC, Ontario, Quebec), and calendar time (based upon specimen collection date, bi-weekly except weeks 07 to 09). TSV refers to interval between 2025/26 COVID-19 vaccination and acute respiratory illness onset among vaccinated participants, except those for whom onset date is unknown, assigned instead based on specimen collection date (n=9).

References

1. National Advisory Committee on Immunization (NACI). Statement on seasonal influenza vaccines for 2025-26 [Internet]. Ottawa: Public Health Agency of Canada; 2025 [cited 2026 Mar 28]. Available from: https://www.canada.ca/en/public-health/services/publications/vaccines-immunization/national-advisory-committee-immunization-statement-seasonal-influenza-vaccines-2025-2026.html

2. O'Toole Á, Scher E, Underwood A, Jackson B, Hill V, McCrone JT, et al. Assignment of epidemiological lineages in an emerging pandemic using the pangolin tool. Virus Evol. 2021;7(2):veab064.

3. Zhang L, Chen N, Eichmann A, Nehlmeier I, Moldenhauer AS, Stankov MV, et al. Epidemiological and virological update on the emerging SARS-CoV-2 variant BA.3.2. The Lancet Infectious Diseases. 2026 Jan;26(1):e1–2.

4. Mila Shakya Dp, Kevin C. Ma P, Laura J. Hughes P, Casey Smith MS, Lydia J. Atherton DVM, Alexandria B. Boehm P, et al. Early Detection and Surveillance of the SARS-CoV-2 Variant BA.3.2 — Worldwide, November 2024–February 2026. MMWR Morb Mortal Wkly Rep [Internet]. 2026 [cited 2026 Mar 23];75. Available from: https://www.cdc.gov/mmwr/volumes/75/wr/mm7510a1.htm

5. Freed N, Murphy L, Schwessinger B, Silander O. 'Midnight' SARS-CoV2 genome sequencing protocol using 1200bp amplicon primer set v2 and the Nanopo... 2023 Apr 14 [cited 2026 Apr 24]; Available from: https://www.protocols.io/view/34-midnight-34-sars-cov2-genome-sequencing-protoc-14egn2q2yg5d/v1

6. Hickman R, Nguyen J, Lee TD, Tyson JR, Azana R, Tsang F, et al. Rapid, high-throughput, cost-effective whole-genome sequencing of SARS-CoV-2 using a condensed library preparation of the Illumina DNA Prep kit. Journal of Clinical Microbiology. 2024 Feb 5;62(3):e00103-22.

7. ARTIC Network. SARS-CoV-2 version 5.4.2 Scheme release [Internet]. ARTIC Real-time Genomic Surveillance. 2023 [cited 2026 Apr 24]. Available from: https://community.artic.network/t/scheme-release-artic-sars-cov2-400-v5-4-2/546

8. Quick J. nCoV-2019 sequencing protocol V.1. 2020 Jan 22 [cited 2026 Apr 24]; Available from: https://www.protocols.io/view/ncov-2019-sequencing-protocol-bp2l6n26rgqe/v1

9. Illumina. Illumina CovidSeq Test Instructions for Use. 2021.

10. Bourgey M, Dali R, Eveleigh R, Chen KC, Letourneau L, Fillon J, et al. GenPipes: an open-source framework for distributed and scalable genomic analyses. Gigascience. 2019 Jun 1;8(6):giz037.

11. Rambaut A, Holmes EC, O'Toole Á, Hill V, McCrone JT, Ruis C, et al. A dynamic nomenclature proposal for SARS-CoV-2 lineages to assist genomic epidemiology. Nat Microbiol. 2020 Nov 1;5(11):1403–7.

12. Shu Y, McCauley J. GISAID: Global initiative on sharing all influenza data – from vision to reality. Euro Surveill. 2017 Mar 30;22(13):30494.
